# Saddle-shaped tetraphenylenes with peripheral gallic esters displaying columnar mesophases

**DOI:** 10.3762/bjoc.5.57

**Published:** 2009-10-21

**Authors:** Eugen Wuckert, Constanze Hägele, Frank Giesselmann, Angelika Baro, Sabine Laschat

**Affiliations:** 1 Institut für Organische Chemie, Universität Stuttgart, Pfaffenwaldring 55, D-70569 Stuttgart, Germany; 2 Institut für Physikalische Chemie, Universität Stuttgart, Pfaffenwaldring 55, D-70569 Stuttgart, Germany

**Keywords:** columnar mesophases, discotic liquid crystals, tetraphenylene

## Abstract

Tetraphenylenes **2** with eight peripheral gallic esters were prepared in two steps from octamethoxytetraphenylene **1** in 19–72% yield. Investigation of the mesomorphic properties of **2** by DSC, POM and X-ray diffraction revealed that derivatives **2a**–**d** with short alkoxy chain lengths (C_5_–C_8_) did not show any mesomorphic properties, whereas compounds **2e**–**i** with C_9_–C_13_ chains displayed rectangular columnar mesophases and compounds **2j**–**l** with C_14_–C_16_ chains displayed hexagonal columnar mesophases. Furthermore an anomalous odd-even effect of the clearing points of compounds **2e**–**l** versus chain length was detected.

## Introduction

Columnar liquid crystals have received increasing interest during the last decade due to their 1D charge transport and self-healing properties, which make them particularly promising candidates for organic field effect transistors, organic photovoltaic devices and light emitting diodes [1–3]. Tetraphenylenes, whose saddle-shaped conformation is caused by the anti-aromatic character of the corresponding central flat 8-membered ring [4–6], are suitable building blocks for supramolecular chemistry, asymmetric catalysis and formation of inclusion complexes [3–28]. We have shown that tetraphenylenes with eight peripheral alkoxy or alkanoate chains display thermotropic columnar and smectic mesophases [29–30]. Furthermore, anomalous odd-even effects were discovered for these discotic tetraphenylenes, i.e. the ascending and descending transition temperatures with increasing numbers of methylene groups in the side chain exhibit an inversion of this alternation between *n* = 12 and *n* = 14 homologues [31]. In order to explore whether this anomalous odd-even effect is a more general phenomenon, the corresponding gallic ester-substituted tetraphenylenes were prepared and their liquid crystalline properties were investigated. In addition, we were curious about the mesophase types, because tetraphenylenes with peripheral alkoxy or alkanoate chains displayed smectic mesophases in addition to columnar phases, whereas the corresponding tetraphenylenes with *p*-alkoxybenzoate substituents displayed only columnar mesophases even with long chain lengths [29–31]. Thus, we anticipated that the presence of the sterically demanding gallic esters in the periphery of the tetraphenylene can be accommodated much better by columnar packing as compared to a smectic layer structure. The results are discussed below.

## Results and Discussion

**Scheme 1:**
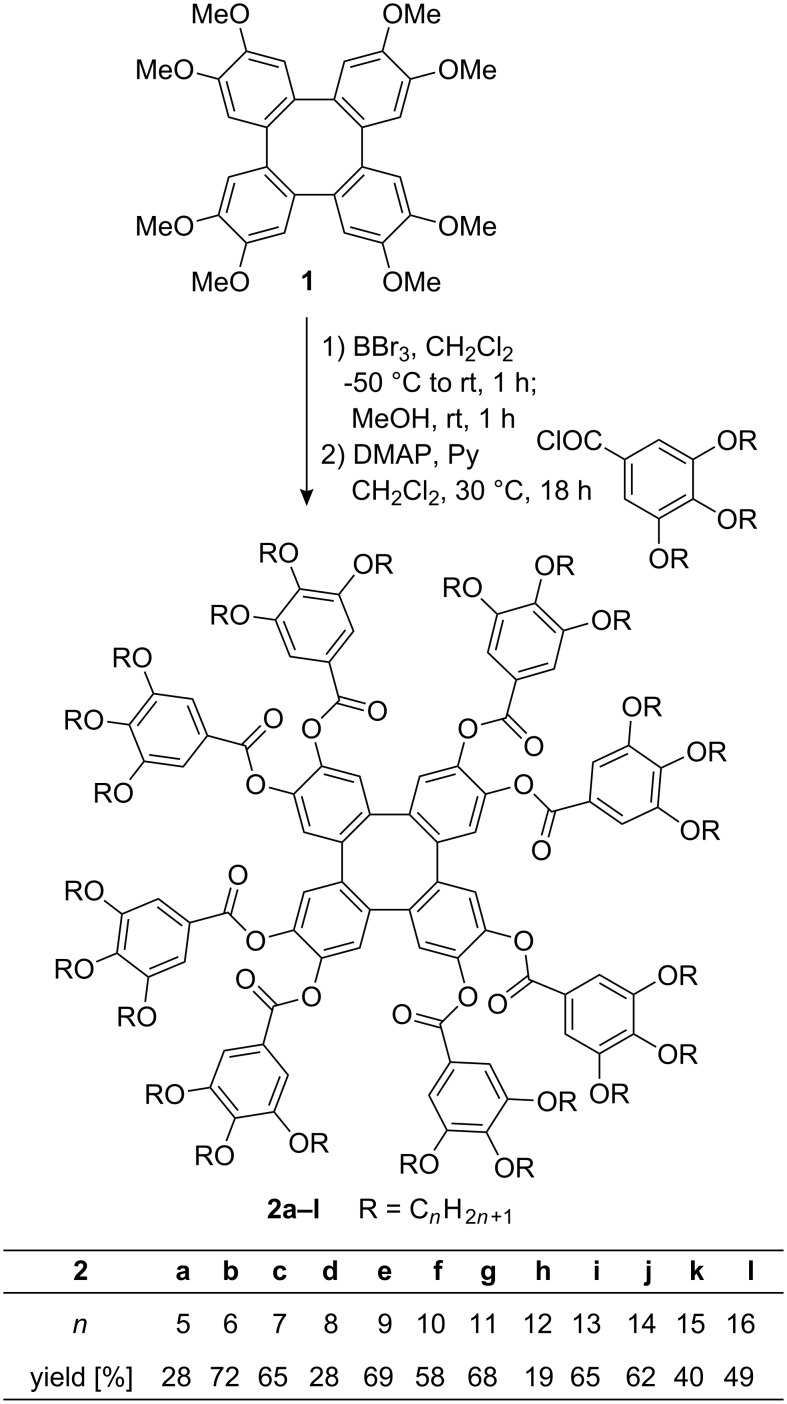
Synthesis of tetraphenylenes **2**.

The synthesis started from the known octamethoxytetraphenylene **1** [4,19–20,29–31], which was demethylated with boron tribromide in CH_2_Cl_2_ at −50 °C to room temp. followed by hydrolysis with MeOH (Scheme 1). Subsequent treatment with gallic acid chlorides in the presence of catalytic amounts of DMAP in pyridine/CH_2_Cl_2_ yielded after aqueous workup and chromatographic purification the desired gallic ester-substituted tetraphenylenes **2a**–**l** in 19–72%. In some cases purification turned out to be rather tedious resulting in decreased yields.

Mesomorphic properties of compounds **2** were studied by differential scanning calorimetry (DSC), polarizing optical microscopy (POM) and X-ray diffraction (WAXS, SAXS). The DSC results are summarized in Table 1.

**Table 1:** Phase transition temperatures [°C] and (enthalpies [kJ/mol])^a^.

**2**	*n*	Cr_1_		Cr_2_		Cr_3_		Col		I	

**a**	5	•	51 (10.1)	•	79 (4.4)	•	191 (15.3)	--		•	2. heating
**b**	6	•	46 (8.3)	•	69 (7.4)	•	142 (11.2)	--		•	2. heating
**c**	7	•	10 (1.7)	•	41 (0.6)	•	55 (0.6)	--		•	2. heating
**d**	8	•	4 (15.0)	•	41 (27.7)	•	62 (7.2)	--		•	2. heating
**e**	9	•	−6 (11.6)	•	35 (4.2)	--		•	37 (3.8)	•	2. heating
**e**	9	•	1 (−2.7)	•	11 (−0.8)	--		•	26 (−5.3)	•	2. cooling
**f**	10	•	7 (4.1)	•	40 (12.5)	--		•	46 (5.6)	•	2. heating
**f**	10	•	24 (−7.9)	--		--		•	39 (−5.7)	•	2. cooling
**g**	11	•	29 (7.4)	--		--		•	43 (1.0)	•	2. heating
**g**	11	•	20 (−7.6)	--		--		•	40 (−1.1)	•	2. cooling
**h**	12	•	3 (19.4)	--		--		•	36 (10.3)	•	2. heating
**h**	12	•	0 (−18.6)	--		--		•	33 (−9.7)	•	2. cooling
**i**	13	•	16 (18.5)	--		--		•	33 (11.9)	•	2. heating
**i**	13	•	13 (−18.9)	--		--		•	28 (−13.4)	•	2. cooling
**j**	14	•	16 (9.6)	--		--		•	41 (2.5)	•	2. heating
**j**	14	•	16 (−5.8)	--		--		•	29 (−1.8)	•	2. cooling
**k**	15	•	25 (8.0)	--		--		•	36 (20.1)	•	2. heating
**k**	15	•	20 (−10.3)	--		--		•	32 (−16.2)	•	2. cooling
**l**	16	--		--		--		•	41 (36.2)	•	2. heating
**l**	16	•	22 (−5.2)	--		--		•	36 (−17.3)	•	2. cooling

^a^Cr crystalline, Col columnar, I isotropic; • phase was observed, -- phase was not observed; heating/cooling rate 10 K/min for **2a**–**e**,**i**,**j**, 5 K/min for **2f**–**h**,**k**,**l**.

**Figure 1:**
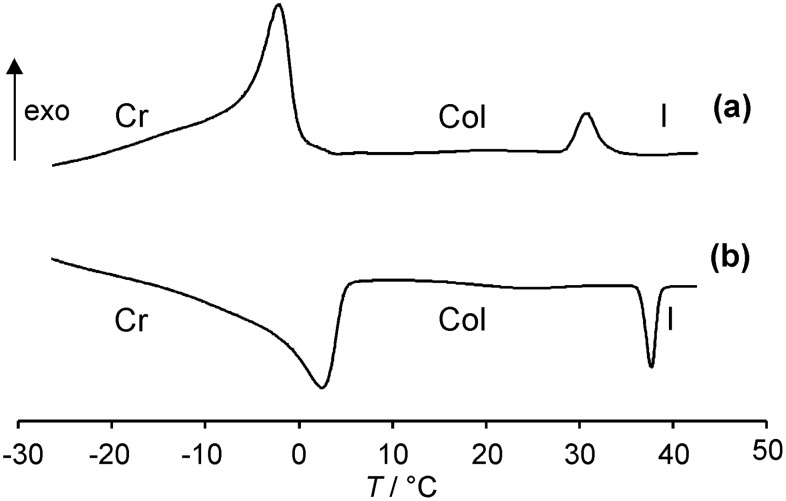
DSC traces of compound **2h** during (a) second cooling and (b) second heating (heating/cooling rate 5 K/min).

While compounds **2a**–**d** with chain lengths up to C_8_ showed only crystal to crystal transitions and isotropic melting, tetraphenylenes **2e**–**l** with chain lengths between C_9_ and C_16_ displayed enantiotropic mesomorphism. For compounds **2e**,**f** additional crystal to crystal transitions were detected. A typical DSC curve is shown in Figure 1. Thus tetraphenylene **2h** with dodecyl chains displays a melting transition at 3 °C and a clearing transition at 36 °C upon second heating. Upon cooling an isotropic to mesophase transition at 33 °C and a crystallization peak at 0 °C were detected. The hysteresis phenomena observed for some compounds are probably due to supercooling, which is common for such highly viscous materials.

**Figure 2:**
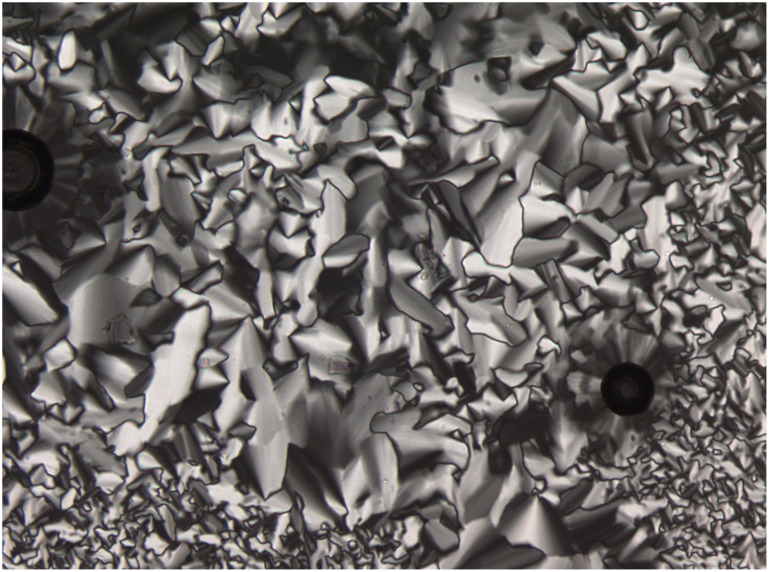
Texture of **2h** under the POM at 25 °C upon cooling from the isotropic liquid (heating/cooling rate 5 K/min; magnification 100×).

POM investigations revealed focal conic and fan-shaped textures typical for columnar mesophases (Figure 2).

However, clear evidence was possible only by XRD data, which are summarized in Table 2. While rectangular columnar mesophases were observed for tetraphenylenes **2e**–**i** with shorter chains (*n* = 9–13) (shown for **2f** in Figure S1 in the Supporting Information), hexagonal columnar mesophases were found for the long chain derivatives **2j**–**l** (*n* = 14–16) (shown for **2j** in Figure S2 in the Supporting Information). Indeed, as expected the columnar mesophase seems to accommodate the eight bulky gallic esters much better than the smectic layer structure.

**Table 2:** X-Ray diffraction data for compounds **2e**–**l**^a^.

**2**	*T* [°C]	θ [°]	*d*_obs_ [Å]	*hk*	*d*_calc_ [Å]	Mesophase parameters

**2e**	20	1.71	25.9	(20)	25.9	Col_r_
		2.93	15.1	(11)	15.1	*a* = 51.8 Å
		3.33	13.3	(21)	13.5	*b* = 15.8 Å
**2f**	32	1.73	25.6	(20)	18.4	Col_r_
		2.32	19.0	(11)	19.0	*a* = 51.2 Å
		3.34	13.2	(31)	13.1	*b* = 20.5 Å
**2g**	15	1.60	27.7	(20)	27.7	Col_r_
		2.17	20.4	(11)	20.4	*a* = 55.4 Å
		3.14	14.1	(31)	14.1	*b* = 21.9 Å
**2h**	20	1.51	29.3	(20)	29.3	Col_r_
		2.25	19.6	(11)	19.6	*a* = 58.6 Å
						*b* = 20.8 Å
**2i**	25	1.47	30.1	(20)	30.1	Col_r_
		2.28	19.4	(11)	19.4	*a* = 60.3 Å
						*b* = 20.5 Å
**2j**	30	1.46	30.3	(10)	30.6	Col_h_
		2.47	17.9	(11)	17.7	*a* = 35.3 Å
**2k**	20	1.40	31.6	(10)	31.9	Col_h_
		2.39	18.5	(11)	18.4	*a* = 36.9 Å
		2.75	16.1	(20)	16.0	
		3.65	12.1	(21)	12.1	
**2l**	20	1.35	32.8	(10)	33.1	Col_h_
		2.32	19.0	(11)	19.1	*a* = 38.2 Å
		3.50	12.6	(21)	12.5	

^a^Diffraction angle θ; observed and calculated diffraction spacings *d*_obs_ and *d*_calc_; Miller indices *hk*.

**Figure 3:**
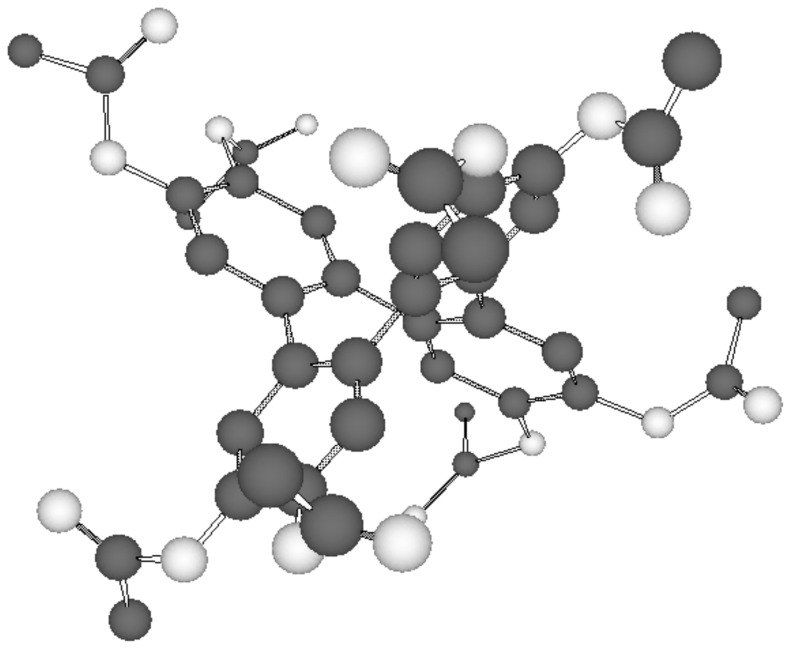
Molecular modelling of the saddle-shaped tetraphenylene (octakis)acyl core unit of **2** [35].

The crossover from rectangular columnar to hexagonal columnar mesophases with increasing chain lengths has been also observed in other columnar systems [32–33] and has been attributed to the enhanced core–core interaction necessary for the formation of the Col_r_ phases [34]. According to molecular modelling [35] and comparison with the XRD data each disk within the hexagonal and rectangular columnar pattern is occupied by one tetraphenylene molecule. For better visibility only the modelled tetraphenylene (octakis)acyl core unit is shown in Figure 3, which reveals the saddle shape.

**Figure 4:**
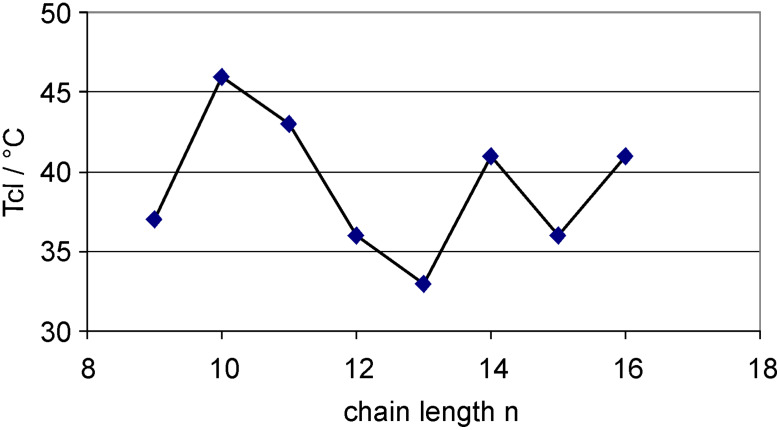
Clearing temperatures *T*_cl_ [°C] of tetraphenylenes **2e**–**l** as a function of the chain lengths *n*.

Next, the clearing points of mesogenic compounds **2e**–**l** were plotted against the chain lengths *n* (Figure 4). An anomalous odd-even effect can be seen, which inverts at *n* = 11.

**Figure 5:**
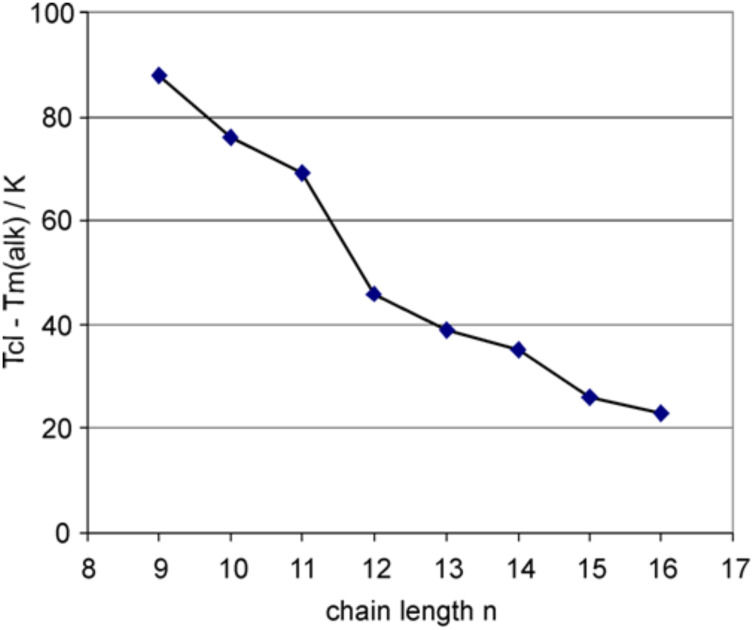
The differences between the clearing temperatures *T*_cl_ [°C] of tetraphenylenes **2** and the melting points *T*_m_^alk^ of the *n*-alkanes exhibit an almost normal odd-even effect without any inversion of the alteration.

For the previously studied tetraphenylene derivatives with alkoxy, alkanoate and *p*-alkoxybenzoate chains the inversion was observed at *n* = 12–13 [31]. Although in all four cases an anomalous odd-even effect is present, the chain length dependence of the clearing temperatures differs somewhat. For derivatives with alkoxy or alkanoate chains directly attached to the tetraphenylene unit, the oxygen atom is part of the chain and thus odd carbon chains are actually even-numbered. They should thus have an elongated shape which should lead to a higher degree of orientational order and hence a higher clearing temperature than the odd-numbered chains (including oxygen), i.e. those with an even-number of carbon atoms. The data in Figure 4 suggest that for 3,4,5-trialkoxygallic esters **2** this argument does not hold and the orientational order and hence the clearing temperature is determined both by the alkoxy chain lengths as well as the rigid gallic acid moiety. In order to eliminate the influence of the alkyl side chain on the odd-even effect of the tetraphenylenes the melting temperatures *T*_m_^alk^ of *n*-alkanes were subtracted from the clearing points *T*_cl_ of the respective tetraphenylenes **2** (Figure 5). An almost regular effect could be seen. Thus it seems that transition temperatures are also governed by the influence of the gallic ester moiety on the dynamic properties of the alkyl tails.

## Conclusion

In conclusion, only columnar mesophases have been found for gallic ester-substituted tetraphenylenes **2e**–**l** with a minimum chain length of *n* = 9. An anomalous odd-even effect was detected, in which the alternation of the melting transition inverses at *n* = 11. The results agree with previous findings, and suggest that the anomalous odd-even effect is a more general phenomenon than previously thought. Investigations to extend this concept to other classes of liquid crystals are currently in progress.

## Experimental

### General

Melting points were measured on a Mettler Toledo DSC822 and are uncorrected. NMR spectra were recorded on a Bruker Avance 300 and Avance 500 spectrometer. FT-IR spectra were recorded on a Bruker Vektor22 spectrometer with MKII Golden Gate Single Reflection Diamant ATR system. Mass spectra were recorded on a Finnigan MAT 95 and a Varian MAT 711 apparatus. Small-angle scattering data from unaligned samples (filled into Mark capillary tubes of 0.7 mm diameter) were obtained using a Kratky compact camera (A. Paar) provided with a temperature controller (A. Paar) and a one-dimensional electronic detector (M. Braun). Aligned samples were exposed in a home-made flat-film camera and the 2D diffraction patterns recorded with an imaging plate detector (Fuji BAS SR). In the flat-film camera, the sample was placed in a small hole of a brass block, the temperature of which was controlled by a Lakeshore controller and kept in a 1.5 T magnetic field for alignment.

Differential scanning calorimetry (DSC) was performed using a Mettler Toledo DSC822, and polarizing optical microscopy (POM) using an Olympus BX50 polarizing microscope combined with a Linkam LTS350 hot stage and a Linkam TP93 central processor. Flash chromatography was performed using Kieselgel 60, 40–63 μm (Fluka). All solvents were dried, and reactions were performed in dried glassware. The used petroleum ether (PE) had a boiling range of 30–75 °C. Octamethoxytetraphenylene **1** was prepared as described in ref. [31].

### General procedure for the preparation of heptakis[(3,4,5-trialkoxybenzoyl)oxy]tetra-phenylen-2-yl 3,4,5-trialkoxybenzoates (**2**)

To a solution of octamethoxytetraphenylene **1** (0.14 g, 0.25 mmol) in CH_2_Cl_2_ (2 mL) were added BBr_3_ (2.2 mL, 2.2 mmol, 1 M solution in CH_2_Cl_2_) at −50 °C and the mixture was stirred for 1 h at room temp. The solvent was removed in vacuo and the residue was dissolved in degassed MeOH (5 mL) for 1 h and evaporated. The residue was dissolved in CH_2_Cl_2_ (2 mL), treated with DMAP (4 mg, 0.03 mmol) and pyridine (1 mL) and gallic acid chloride (5 mmol) were added dropwise. After stirring overnight at 30 °C, the mixture was diluted with CH_2_Cl_2_ (10 mL), hydrolyzed with 2 M HCl and the layers separated. The aqueous layer was extracted with CH_2_Cl_2_ (2 × 10 mL), the organic layers were washed with sat. NaHCO_3_ (10 mL), H_2_O (10 mL), dried over MgSO_4_ and concentrated in vacuo. The crude product was purified by column chromatography on SiO_2_ (hexanes/ethyl acetate 20 : 1) to yield colorless waxy solids.

### 3,6,7,10,11,14,15-Heptakis[(3,4,5-dodecyloxybenzoyl)oxy]tetraphenylen-2-yl 3,4,5-tridodecyloxybenzoate (**2h**)

270 mg (19%) of a colorless solid. ^1^H NMR (500 MHz, CDCl_3_): δ = 0.85–0.89 (m, 72H), 1.25–1.49 (m, 432H), 1.70–1.75 (m, 48H), 3.79–3.86 (m, 32H), 3.98 (t, *J* = 6.5 Hz, 16H), 7.24 (s, 16H), 7.39 (s, 8H) ppm. ^13^C NMR (125 MHz, CDCl_3_): δ = 14.1, 22.7, 26.1, 26.2, 29.4, 29.4, 29.5, 29.6, 29.7, 29.7, 29.8, 29.8, 30.4, 32.0, 69.0, 73.5, 108.3, 123.0, 124.5, 138.1, 142.0, 143.0, 152.9, 163.8 ppm. FT-IR (ATR): ν = 2921 (vs), 2852 (s), 1976 (w), 1743 (m), 1585 (m), 1499 (w), 1466 (w), 1430 (m), 1390 (w), 1335 (s), 1291 (w), 1240 (w), 1190 (s), 1114 (s), 947 (w), 802 (w), 748 (w), 623 (m), 546 (w) cm^−1^. UV/VIS (*n*-hexane): λ_max_ (lg ε _max_) = 275 (5.15), 214 (5.60) nm. C_368_H_624_O_40_ (5688.9) calcd. C 77.69, H 11.06; found: C 77.74, H 10.95.

## Supporting Information

Supporting information includes experimental and spectroscopic data for compounds **2a**–**f**, **2g**–**l**, and X-ray diffraction measurements of derivatives **2f**,**j**.

File 1 Analytical data of compounds **2a**–**f**, **2g**–**l**.
