# Supplementary material for: Saddle-shaped tetraphenylenes with peripheral gallic esters displaying columnar mesophases
Source: Beilstein J Org Chem. 2009 Oct 21;5:57. doi: 10.3762/bjoc.5.57 (PMC2813710; doi:10.3762/bjoc.5.57)
Supplement: File 1 — Analytical data of compounds 2a–f, 2g–l. [file Beilstein_J_Org_Chem-05-57-s001.pdf]

Saddle-shaped tetraphenylenes with peripheral gallic esters displaying columnar mesophases

Eugen Wuckert[1], Constanze Hägele[2], Frank Giesselmann[2], Angelika Baro[1] and Sabine Laschat[*1]

[1]Institut für Organische Chemie, Universität Stuttgart, Pfaffenwaldring 55, D-70569 Stuttgart, Germany and [2]Institut für Physikalische Chemie, Universität Stuttgart, Pfaffenwaldring 55, D-70569 Stuttgart, Germany

Email: Frank Giesselmann - frank.giesselmann@ipc.uni-stuttgart.de;

Sabine Laschat[*] - sabine.laschat@oc.uni-stuttgart.de

[*] Corresponding author

Supporting Information

Analytical data of tetraphenylenes **2a**–**f**, **2g**–**l**.

3,6,7,10,11,14,15-Heptakis[(3,4,5-pentyloxybenzoyl)oxy]tetraphenylen-2-yl 3,4,5-tri-pentyloxybenzoate (2a)

233 mg (28 %) of a colorless solid. ^{1}H NMR (500 MHz, $CDCl_3$): δ = 0.89–0.93 (m, 72H), 1.32–1.49 (m, 96H), 1.71–1.78 (m, 48H), 3.82-3.86 (m, 32H), 3.99 (t, *J* = 6.5 Hz, 16H), 7.26 (s, 16H), 7.39 (s, 8H) ppm. ^{13}C NMR (125 MHz, $CDCl_3$): δ = 14.0, 14.1, 22.4, 22.5, 28.2, 28.2, 29.0, 30.0, 69.0, 73.4, 108.3, 123.0, 124.5, 138.1, 142.0, 143.0, 152.9, 163.8 ppm. FT-IR (ATR): ν = 2954 (m), 2930 (s), 2870 (m), 2167 (w), 1966 (w), 1740 (s), 1585 (m), 1499 (w),

1467 (w), 1429 (m), 1389 (w), 1333 (vs), 1291 (w), 1240 (w), 1187 (vs), 1109 (vs), 1046 (w), 978 (m), 944 (w), 925 (w), 886 (w), 815 (w), 747 (m), 696 (w), 579 (w) cm^{-1}. UV–vis (*n*-hexane): λ_{max} (lg ε_{max}) = 276 (5.12), 216 (5.51) nm. $C_{200}H_{288}O_{40}$ (3332.4) calcd. C 72.08, H 8.71; found: C 71.90, H 8.73.

3,6,7,10,11,14,15-Heptakis[(3,4,5-hexyloxybenzoyl)oxy]tetraphenylen-2-yl 3,4,5-tri-hexyloxybenzoate (2b)

660 mg (72 %) of a colorless solid. ^{1}H NMR (500 MHz, $CDCl_3$): δ = 0.87–0.91 (m, 72H), 1.30–1.48 (m, 144H), 1.70–1.77 (m, 48H), 3.82–3.86 (m, 32H), 3.99 (t, *J* = 6.6 Hz, 16H), 7.25 (s, 16H), 7.39 (s, 8H) ppm. ^{13}C NMR (125 MHz, $CDCl_3$): δ = 14.0, 14.1, 22.6, 22.7, 25.7, 25.8, 29.2, 30.3, 31.6, 31.7, 69.0, 73.5, 108.3, 123.0, 124.5, 138.1, 142.0, 143.0, 152.9, 163.8 ppm. FT-IR (ATR): ν = 2953 (m), 2927 (s), 2858 (m), 2165 (w), 1966 (w), 1741 (s), 1585 (m), 1499 (w), 1467 (w), 1429 (m), 1390 (w), 1333 (vs), 1291 (w), 1240 (w), 1187 (vs), 1110 (vs), 983 (w), 919 (w), 891 (w), 860 (w), 747 (s), 591 (w) cm^{-1}. UV–vis (*n*-hexane): λ_{max} (lg ε_{max}) = 276 (5.09), 216 (5.50) nm. $C_{224}H_{336}O_{40}$ (3669.0 g/mol) calcd. C 73.33, H 9.23; found: C 73.08, H 9.15.

3,6,7,10,11,14,15-Heptakis[(3,4,5-heptyloxybenzoyl)oxy]tetraphenylen-2-yl 3,4,5-tri-heptyloxybenzoate (2c)

662 mg (65 %) of a colorless solid. ^{1}H NMR (500 MHz, $CDCl_3$): δ = 0.86–0.90 (m, 72H), 1.26–1.48 (m, 192H), 1.71–1.77 (m, 48H), 3.81–3.86 (m, 32H), 3.99 (t, *J* = 6.6 Hz, 16H), 7.25 (s, 16H), 7.40 (s, 8H) ppm. ^{13}C NMR (125 MHz, $CDCl_3$): δ = 14.1, 14.1, 22.6, 22.7, 20.0, 26.1, 29.1, 29.2, 29.3, 30.4, 31.8, 31.9, 69.0, 73.5, 108.3, 123.0, 124.5, 138.1, 142.0, 143.0, 152.9, 163.8 ppm. FT-IR (ATR): ν = 2953 (m), 2924 (s), 2855 (m), 2166 (w), 1966 (w), 1742 (s), 1585 (m), 1499 (w), 1467 (w), 1429 (m), 1390 (w), 1333 (vs), 1291 (w), 1272 (w), 1187 (vs), 1111 (vs), 986 (w), 934 (w), 861 (w), 817 (w), 747 (m), 639 (m), 619 (m)

cm^{-1}. UV–vis (*n*-hexane): λ_{max} (lg ε_{max}) = 276 (5.13), 216 (5.53) nm. $C_{249}H_{388}O_{40}$ (4075.8) calcd. C 74.36, H 9.72; C 74.26, H 9.57.

3,6,7,10,11,14,15-Heptakis[(3,4,5-octyloxybenzoyl)oxy]tetraphenylen-2-yl 3,4,5-tri-octyloxybenzoate (2d)

304 mg (28 %) of a colorless solid. ^{1}H-NMR (500 MHz, $CDCl_3$): δ = 0.86–0.89 (m, 72H), 1.27–1.48 (m, 240H), 1.71–1.76 (m, 48H), 3.81–3.85 (m, 32H), 3.98 (t, J = 6.6 Hz, 16H), 7.25 (s, 16H), 7.40 (s, 8H) ppm. ^{13}C-NMR (125 MHz, $CDCl_3$): δ = 14.1, 22.7, 22.7, 26.1, 26.1, 29.3, 29.4, 29.4, 29.5, 30.4, 31.9, 31.9, 69.0, 73.5, 108.3, 123.0, 124.5, 138.1, 142.0, 143.0, 152.9, 163.8 ppm. FT-IR (ATR): ν = 2922 (vs), 2854 (s), 1742 (s), 1585 (m), 1499 (w), 1467 (w), 1430 (m), 1390 (w), 1334 (s), 1291 (w), 1274 (w), 1192 (vs), 1113 (vs), 986 (w), 946 (w), 890 (w), 748 (w), 632 (s), 530 (s) cm^{-1}. UV–vis (*n*-hexane): λ_{max} (lg ε_{max}) = 277 (5.11), 216 (5.49) nm. $C_{270}H_{428}O_{40}$ (4342.3) calcd. C 75.17, H 10.00; found: C 75.36, H 9.88.

3,6,7,10,11,14,15-Heptakis[(3,4,5-nonyloxybenzoyl)oxy]tetraphenylen-2-yl 3,4,5-tri-nonyloxybenzoate (2e)

807 mg (69 %) of a colorless solid. ^{1}H-NMR (500 MHz, $CDCl_3$): δ = 0.85-0.89 (m, 72H), 1.27–1.47 (m, 288H), 1.71–1.76 (m, 48H), 3.80–3.85 (m, 32H), 3.98 (t, J = 6.5 Hz, 16H), 7.26 (s, 16H), 7.39 (s, 8H) ppm. ^{13}C-NMR (125 MHz, $CDCl_3$): δ = 14.1, 22.7, 22.7, 26.1, 26.1, 29.3, 29.4, 29.4, 29.5, 30.4, 31.9, 31.9, 69.0, 73.5, 108.3, 123.0, 124.5, 138.1, 142.0, 143.0, 152.9, 163.8 ppm. FT-IR (ATR): ν = 2921 (s), 2853 (s), 1744 (m), 1586 (m), 1500 (w), 1467 (w), 1431 (m), 1390 (w), 1336 (s), 1292 (w), 1272 (w), 1194 (m), 1115 (m), 985 (w), 891 (w), 748 (w), 632 (vs), 536 (vs) cm^{-1}. UV–vis (*n*-hexane): λ_{max} (lg ε_{max}) = 276 (5.11), 216 (5.51) nm. $C_{296}H_{480}O_{40}$ (4679.0) calcd. C 75.98, H 10.34; found: C 76.15, H 10.30.

3,6,7,10,11,14,15-Heptakis[(3,4,5-decyloxybenzoyl)oxy]tetraphenylen-2-yl 3,4,5-tri-decyloxybenzoate (2f)

727 mg (58 %) of a colorless solid. ^{1}H NMR (500 MHz, $CDCl_3$): δ = 0.85–0.89 (m, 72H), 1.26–1.48 (m, 336H), 1.70–1.76 (m, 48H), 3.80–3.85 (m, 32H), 3.98 (t, J = 6.5 Hz, 16H), 7.25 (s, 16H), 7.40 (s, 8H) ppm. ^{13}C NMR (125 MHz, $CDCl_3$): δ = 14.1, 22.7, 22.7, 26.1, 26.1, 29.3, 29.4, 29.4, 29.5, 29.6, 29.6, 29.7, 29.7, 29.7, 29.8, 30.4, 31.9, 31.9, 69.0, 73.5, 108.3, 123.0, 124.5, 138.1, 142.0, 143.0, 152.9, 163.8 ppm. FT-IR (ATR): ν = 2953 (m), 2920 (vs), 2852 (s), 1743 (m), 1586 (m), 1500 (w), 1467 (w), 1431 (m), 1390 (w), 1336 (s), 1116 (m), 983 (w), 924 (w), 890 (w), 748 (w), 632 (vs), 531 (vs) cm^{-1}. UV–vis (*n*-hexane): λ_{max} (lg ε_{max}) = 277 (5.16), 216 (5.57) nm. $C_{320}H_{528}O_{40}$ (5015.6) calcd. C 76.63, H 10.61; found: C 76.53, H 10.53.

3,6,7,10,11,14,15-Heptakis[(3,4,5-undecyloxybenzoyl)oxy]tetraphenylen-2-yl 3,4,5-tri-undecyloxybenzoate (2g)

910 mg (68 %) of a colorless solid. ^{1}H NMR (500 MHz, $CDCl_3$): δ = 0.85–0.89 (m, 72H), 1.26–1.46 (m, 384H), 1.72–1.74 (m, 48H), 3.81–3.83 (m, 32H), 3.98 (t, *J* = 6.5 Hz, 16H), 7.24 (s, 16H), 7.39 (s, 8H) ppm. ^{13}C NMR (125 MHz, $CDCl_3$): δ = 14.1, 22.7, 22.7, 26.1, 26.1, 29.4, 29.4, 29.5, 29.6, 29.7, 29.7, 29.8, 30.4, 32.0, 32.0, 69.0, 73.5, 108.3, 123.0, 124.5, 138.1, 142.0, 143.0, 152.9, 163.8 ppm. FT-IR (ATR): ν = 2921 (vs), 2852 (s), 1744 (m), 1586 (m), 1500 (w), 1467 (w), 1431 (w), 1390 (w), 1336 (m), 1292 (w), 1242 (w), 1193 (m), 1117 (m), 986 (w), 891 (w), 749 (w), 632 (vs), 537 (vs) cm^{-1}. UV–vis (n-Hexan): λ_{max} (lg ε_{max}) = 276 (5.10), 216 (5.49) nm. $C_{344}H_{576}O_{40}$ (5352.2) calcd. C 77.20, H 10.85; found: C 77.29, H 10.77.

3,6,7,10,11,14,15-Heptakis[(3,4,5-tridecyloxybenzoyl)oxy]tetraphenylen-2-yl 3,4,5-tri-tridecyloxybenzoate (2i)

979 mg (65 %) of a colorless solid. ^{1}H NMR (500 MHz, $CDCl_3$): δ = 0.86–0.89 (m, 72H), 1.25–1.49 (m, 480H), 1.71–1.74 (m, 48H), 3.82–3.83 (m, 32H), 3.98 (t, *J* = 6.5 Hz, 16H), 7.24 (s, 16H), 7.39 (s, 8H) ppm. ^{13}C NMR (125 MHz, $CDCl_3$): δ = 14.1, 22.7, 26.1, 26.2, 29.4, 29.4, 29.6, 29.7, 29.7, 29.8, 30.4, 32.0, 69.0, 73.5, 108.3, 123.0, 124.5, 138.1, 142.0, 143.0, 152.9, 163.8 ppm. FT-IR (ATR): ν = 2920 (vs), 2852 (vs), 1744 (m), 1586 (m), 1500 (w), 1487 (w), 1431 (m), 1391 (w), 1336 (m), 1292 (w), 1242 (w), 1194 (m), 1117 (m), 986 (w), 891 (w), 749 (w), 720 (w), 632 (vs) cm^{-1}. UV–vis (*n*-hexane): λ_{max} (lg ε_{max}) = 274 (5.16), 213 (5.70) nm. $C_{392}H_{672}O_{40}$ (6025.5) calcd. C 78.14, H 11.24; found: C 78.23, H 11.19.

3,6,7,10,11,14,15-Heptakis[(3,4,5-tetradecyloxybenzoyl)oxy]tetraphenylen-2-yl 3,4,5-tri-tetradecyloxybenzoate (2j)

986 mg (62 %) of a colorless solid. ^{1}H NMR (500 MHz, $CDCl_3$): δ = 0.86–0.89 (m, 72H), 1.25–1.49 (m, 528H), 1.70–1.75 (m, 48H), 3.78–3.84 (m, 32H), 3.96 (t, *J* = 6.5 Hz, 16H), 7.24 (s, 16H), 7.39 (s, 8H) ppm. ^{13}C NMR (125 MHz, $CDCl_3$): δ = 14.1, 22.7, 26.1, 26.2, 29.4, 29.4, 29.6, 29.7, 29.7, 29.8, 29.8, 29.8, 30.4, 32.0, 69.0, 73.5, 108.3, 123.0, 124.5, 138.1, 142.0, 143.0, 152.9, 163.8 ppm. FT-IR (ATR): ν = 2919 (vs), 2850 (vs), 1743 (m), 1586 (m), 1499 (w), 1467 (w), 1431 (w), 1390 (w), 1335 (s), 1291 (w), 1273 (w), 1241 (w), 1192 (s), 1117 (s), 986 (w), 926 (w), 891 (w), 749 (w), 721 (w), 631 (vs), 539 (vs) cm^{-1}. UV–vis (*n*-hexane): λ_{max} (lg ε_{max}) = 277 (5.10), 217 (5.46) nm. $C_{416}H_{720}O_{40}$ (6362.1) calcd. C 78.53, H 11.41; found: C 78.32, H 11.33.

3,6,7,10,11,14,15-Heptakis[(3,4,5-pentadecyloxybenzoyl)oxy]tetraphenylen-2-yl 3,4,5-tri-pentadecyloxybenzoate (2k)

670 mg (40 %) of a colorless solid. ^{1}H NMR (500 MHz, $CDCl_3$): δ = 0.86–0.89 (m, 72H), 1.25–1.46 (m, 576H), 1.70–1.75 (m, 48H), 3.80–3.84 (m, 32H), 3.97 (t, *J* = 6.5 Hz, 16H), 7.24 (s, 16H), 7.39 (s, 8H) ppm. ^{13}C NMR (125 MHz, $CDCl_3$): δ = 14.1, 22.7, 26.1, 26.2, 29.4, 29.4, 29.6, 29.7, 29.7, 29.8, 29.8, 30.4, 32.0, 69.0, 73.5, 108.3, 123.0, 124.5, 138.1, 142.0, 143.0, 152.9, 163.8 ppm. FT-IR (ATR): ν = 2916 (vs), 2849 (vs), 1966 (w), 1743 (m), 1586 (m), 1499 (w), 1467 (m), 1430 (m), 1389 (w), 1334 (s), 1292 (w), 1240 (w), 1189 (s), 1115 (s), 983 (w), 939 (w), 891 (w), 861 (w), 747 (m), 721 (m), 671 (w), 633 (w), 592 (w) cm^{-1}. UV–vis (*n*-hexane): λ_{max} (lg ε_{max}) = 276 (5.05), 216 (5.50) nm. $C_{440}H_{768}O_{40}$ (6698.8) calcd. C 78.89, H 11.56; found: C 78.67, H 11.51.

3,6,7,10,11,14,15-Heptakis[(3,4,5-hexadecyloxybenzoyl)oxy]tetraphenylen-2-yl 3,4,5-tri-hexadecyloxybenzoate (2l)

862 mg (49 %) of a colorless solid. ^{1}H NMR (500 MHz, $CDCl_3$): δ = 0.86–0.89 (m, 72H), 1.25–1.47 (m, 624H), 1.70–1.75 (m, 48H), 3.80–3.84 (m, 32H), 3.97 (t, *J* = 6.5 Hz, 16H), 7.24 (s, 16H), 7.39 (s, 8H) ppm. ^{13}C NMR (125 MHz, $CDCl_3$): δ = 14.1, 22.7, 26.1, 26.2, 29.4, 29.4, 29.6, 29.7, 29.7, 29.7, 29.8, 29.8, 30.4, 32.0, 69.0, 73.5, 108.3, 123.0, 124.5, 138.1, 142.0, 143.0, 152.9, 163.8 ppm. FT-IR (ATR): ν = 2916 (vs), 2849 (vs), 1743 (m), 1586 (m), 1499 (w), 1467 (w), 1430 (m), 1389 (w), 1334 (s), 1291 (w), 1240 (w), 1190 (s), 1116 (s), 983 (w), 943 (w), 890 (w), 816 (w), 747 (m), 721 (m), 673 (w), 579 (w), 559 (w) cm^{-1}. UV–vis (*n*-hexane): λ_{max} (lg ε_{max}) = 276 (5.08), 216 (5.53) nm. $C_{464}H_{816}O_{40}$ (7035.4) calcd. C 79.21, H 11.69; found: C 79.01, H 11.67.

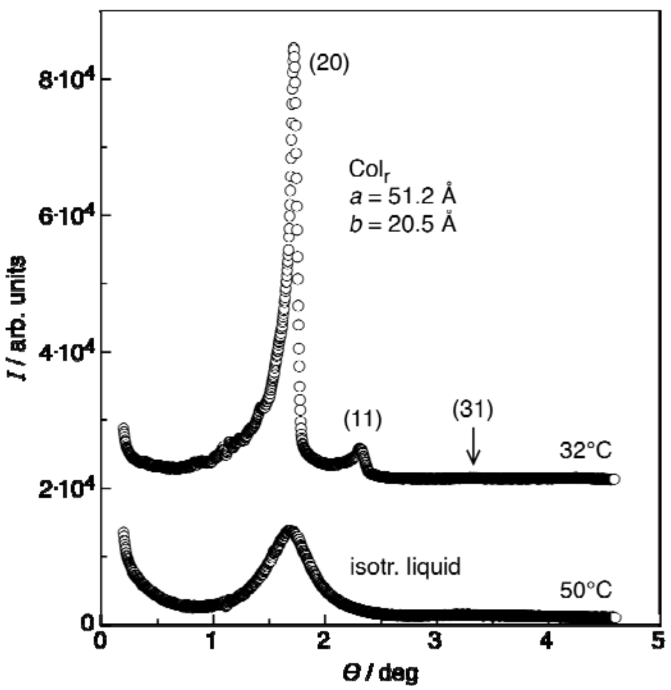

Figure S1: X-ray scattering profile of derivative **2f**.

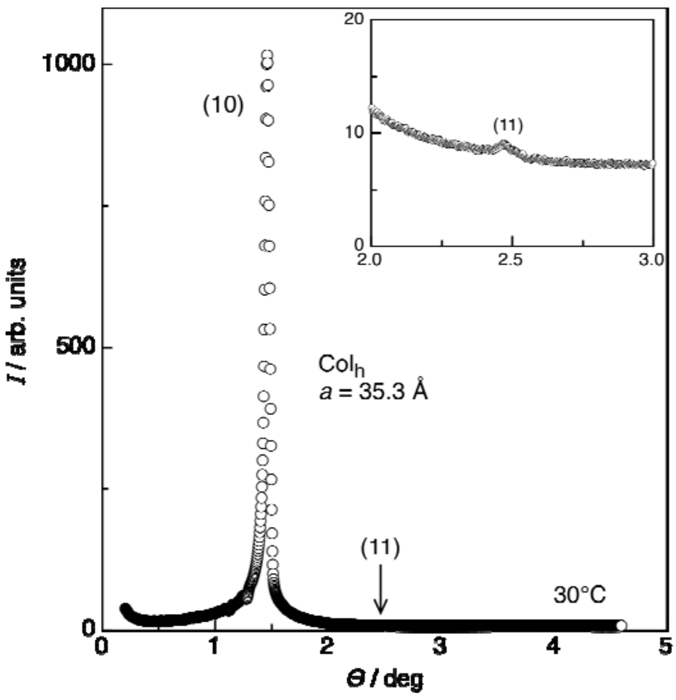

Figure S2: X-ray scattering profile of derivative **2j** at 30 °C.
